# A Case of Solitary Peutz-Jeghers Syndrome Leading to Chronic Small Bowel Obstruction Due to Intussusception From a Large Hamartomatous Polyp

**DOI:** 10.7759/cureus.37679

**Published:** 2023-04-17

**Authors:** Emery Edmondson, Georgios Mihalopulos, Pei E Kwok, Audrey Dellert, Luke Olsen, Zhongqiu J Zhang

**Affiliations:** 1 General Surgery, Waterbury Hospital, Waterbury, USA; 2 Surgery, University of Connecticut School of Medicine, Farmington, USA; 3 Pathology and Laboratory Medicine, Waterbury Hospital, Waterbury, USA; 4 Colorectal Surgery, Waterbury Hosptial, Waterbury, USA

**Keywords:** pjs, intussusception, hamartomatous polyp, small bowel obstruction, solitary peutz-jeghers syndrome

## Abstract

We report a case of a 33-year-old male who presented to the emergency department with a three-day history of severe diffuse abdominal pain associated with anorexia, nausea, and vomiting. Computed tomography (CT) imaging of the abdomen and pelvis revealed a long segment of intussusception in the proximal jejunum and a round lesion along the intussusception with punctate hyperdensities. The patient underwent a diagnostic laparoscopy converted to open small bowel resection and end-to-end anastomosis that demonstrated a pedunculated jejunal mass. The mass was removed, and the pathology revealed a hamartomatous polyp with features of Peutz-Jeghers syndrome (PJS). The patient did not have a family history, previous endoscopic findings, or physical exam findings such as mucocutaneous pigmentation that could be attributed to PJS.

Definitive diagnosis of solitary PJS-type hamartomatous polyps depends on histopathological findings. Genetic analysis for mutations of the PJS susceptible gene, STK11/LB1 located at 19p13.3, as well as loss of heterozygosity at that locus, have been used for the diagnosis of PJS. In patients with large pedunculated hamartomatous polyps, chronic intussusception can occur. If pathology reveals features of Peutz-Jeghers, but the patient lacks the characteristic mucocutaneous pigmentation, family history of PJS, or additional polyps within the GI tract, then solitary PJS may be suspected.

## Introduction

Peutz-Jeghers syndrome (PJS) was first described as a syndrome by Dr. Jan Peutz in 1921 [1]. It is an autosomal dominant polypoid syndrome that manifests as mucocutaneous pigmentation and gastrointestinal hamartomatous polyps. Diagnostic confirmation is based on clinical manifestations and histopathology. Solitary PJS-type hamartomatous polyps are rare and were reported in 1989 by Kuwano et al. [2]. They are regarded as a variant of PJS and may even be considered a different clinical entity. Hamartomatous polyps most commonly occur in the small bowel, colon, and stomach, with 64% being found in the small bowel.

## Case presentation

**Figure 1:**
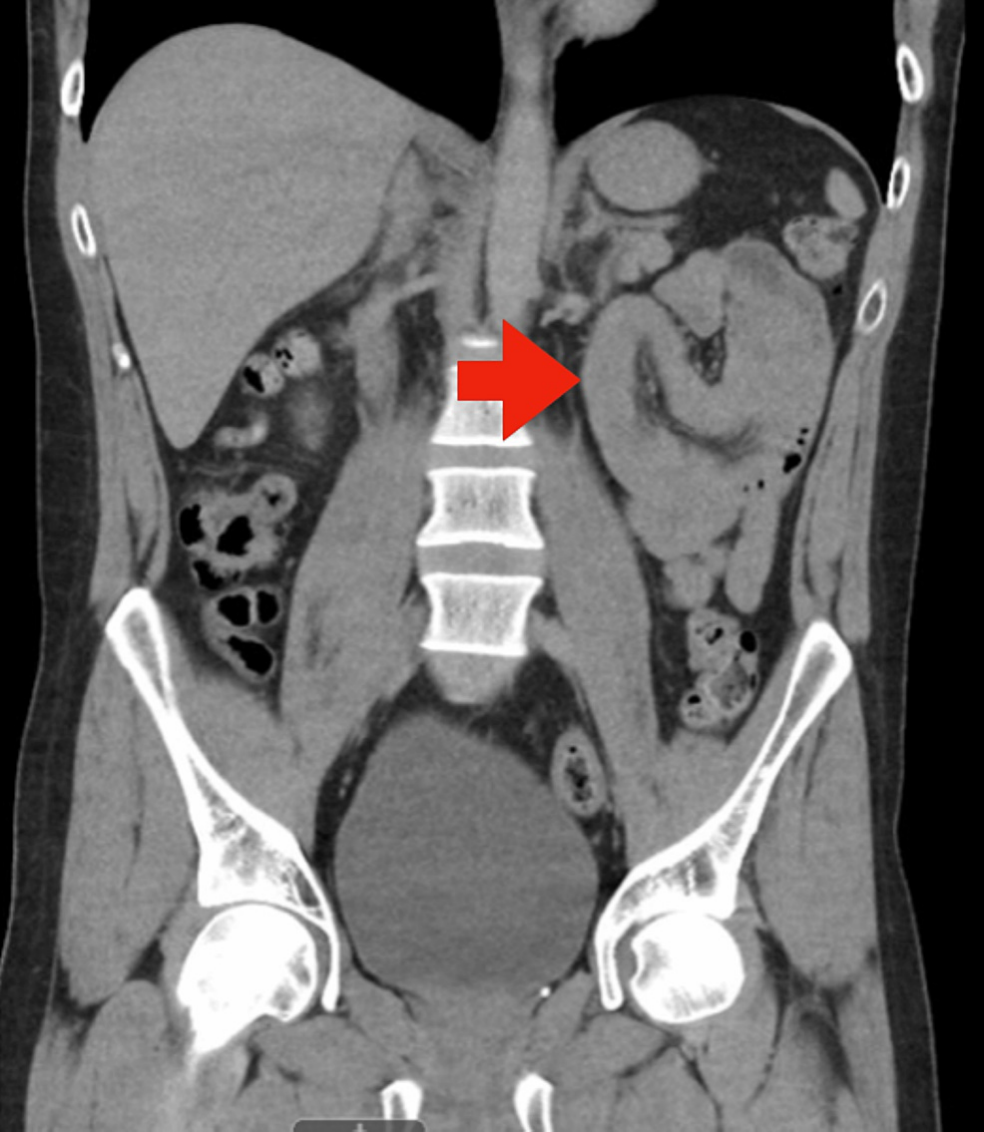
CT abdomen/pelvis without contrast demonstrating intussusception of the proximal jejunum containing a possible round intraluminal mass

This is a case of a 33-year-old male with a medical history of gastritis and diverticulosis and no personal family history of genetic disorders. The patient presented to the emergency department with a three-day history of severe diffused abdominal pain, anorexia, nausea, and vomiting. The patient endured a four-year history of multiple similar episodes that resolved spontaneously. At the time of presentation, the patient was afebrile, normotensive, but with tachycardia of 110. On examination, his abdomen was moderately tender to palpation in all quadrants. No notable abnormalities, such as membranous pigmentation in his oral mucosa, were appreciated. His labs were only remarkable for a leukocytosis of 13.7 thousand/mm^3.^ His Hgb was 15.3 g/dl. CT abdomen and pelvis without contrast demonstrated intussusception of the proximal jejunum containing a possible round intraluminal mass (Figure 1). 

**Figure 2:**
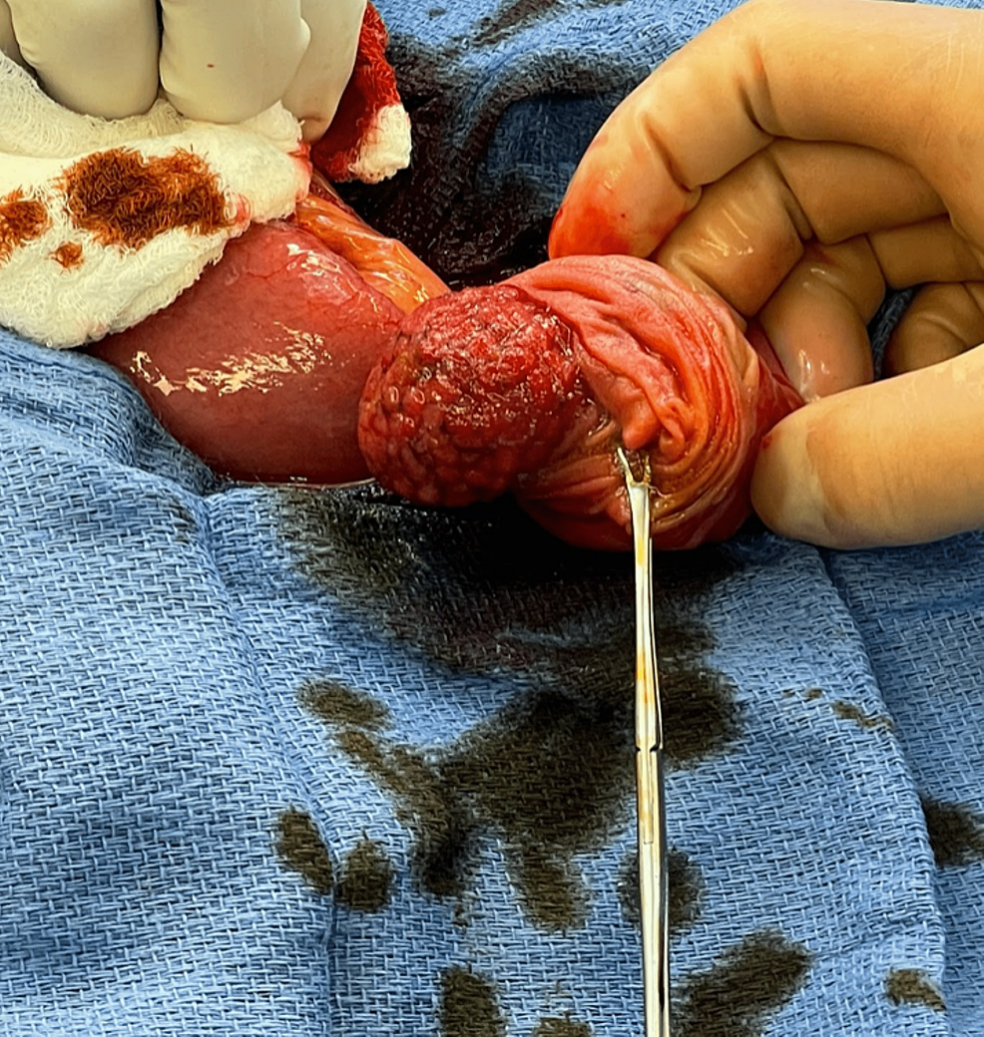
Transected proximal jejunum with an intraluminal pedunculated 3 cm mass

Given the patient's imaging findings and an examination consistent with chronic small bowel obstruction, the patient was made nil per os (NPO), and IV fluids were started. The patient refused nasogastric tube placement. He was then promptly taken to the operating room and underwent a diagnostic laparoscopy which was remarkable for multiple areas of adhesions and a dilated proximal jejunum with an obvious transition point not associated with adhesions and an intra-luminal mass was palpated with laparoscopic graspers. The remainder of the abdomen was inspected laparoscopically, with no further pathology identified. Conversion to a mini-laparotomy was conducted in order to evaluate and palpate the involved segment more thoroughly. The small bowel was eviscerated, and a mass was then palpated in the lumen of the area of the small bowel in question. The mass was intraluminal, mobile, and approximately 3 cm (Figure 2). The jejunum around the mass was incised. This pedunculated mass on the mesenteric side of the jejunum was found approximately 15 cm away from the ligament of Treitz. This portion of the small bowel encompassing the mass was resected and sent to pathology. Given the proximity to the ligament of Treitz, it was decided a side-to-side anastomosis would provide too much tension on the reconstruction. Instead, an end-to-end anastomosis was performed using an EEA 25 stapler (Medtronic, Dublin, Ireland) inserted via a distal enterotomy. 

**Figure 3:**
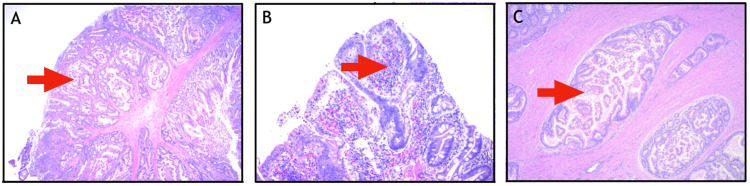
Histological examination of the 3.8 cm polyp Histological examination of the 3.8 cm polyp revealed arborizing fronds supported by broad bands of muscularis mucosa centrally surfaced by small intestinal epithelium (A). Focal low-grade dysplasia is seen (B). Examination of some bands of muscularis mucosae under high magnification reveals prolapse-related misplacement of benign epithelium (pseudoinvasion) (C). (A) Hematoxylin-eosin stain; 2X; (B) 10X; (C) 10X

The patient was discharged on postoperative day three. Histopathology revealed a 3.8 x 2.8 x 2.5 cm friable hamartomatous polyp with features of PJS, focal low-grade dysplasia, and prolapse-type changes negative for high-grade dysplasia or carcinoma (Figure 3). The patient underwent an esophagogastroduodenoscopy and colonoscopy four months postoperatively which was unremarkable.

## Discussion

We report a case of solitary Peutz-Jeghers syndrome and a hamartomatous polyp found in the jejunum of a 33-year-old male who presented with intussusception causing recurrent partial small bowel obstructions. The patient demonstrated no characteristic manifestations of PJS, given the absence of mucocutaneous pigmentation, family history, and multiple gastrointestinal polyps. 

The overwhelming majority of acute small bowel obstructions in developed countries are attributed to adhesions and incarcerated hernias, the majority of which will resolve with non-operative management. However, in the remaining 10% of cases, the etiology is due to other causes, including masses, malignancy, and inflammatory bowel disease [3], which usually requires surgical intervention.

In this patient, the pedunculated polyp produced a lead point, causing a small bowel to small bowel intussusception, and thus, a functional, symptomatic small bowel obstruction that was likely transient due to intermittent reduction of the intussusception. As in this patient, this can produce years of intermittent and recurrent symptoms, all without the typical imaging findings of dilated small bowel, air-fluid levels, and a transition point. This is, therefore, an elusive etiology of bowel obstruction.

Peutz-Jeghers syndrome was first described by Dr. Jan Peutz in 1921 [1]. It is an autosomal dominant polypoid syndrome that manifests as mucocutaneous pigmentation and gastrointestinal hamartomatous polyps [1,4,5]. Diagnostic confirmation is based on clinical manifestations and histopathology. The hamartomatous polyposis syndromes (HPS) encompass Peutz-Jeghers syndrome, Juvenile polyposis syndrome, phosphatase and tensin homolog (PTEN) syndrome (Cowden syndrome, Bannayan-Riley-Ruvalcaba, and Proteus syndrome), as well as hereditary mixed polyposis syndrome. HPS are rare and characterized by hamartomatous polyps within the gastrointestinal tract, along with extra-intestinal manifestations such as mucocutaneous pigmented macules [6]. These disorders are caused by a range of germline mutations, including SMAD4, BMPR1A, and ENG (juvenile polyposis syndrome), and PTEN (Cowden syndrome), as a few examples. In the case of Peutz-Jeghers syndrome, patients inherit a germline mutation in STK11, which is associated with an increased risk of GI, gynecologic, and breast malignancies [6].

Solitary PJS-type hamartomatous polyps are rare and were reported in 1989 by Kuwano et al. [2]. They are regarded as a variant of PJS and may even be considered a different clinical entity [7,8]. In general, hamartomatous polyps most commonly occur in the small bowel, colon, and stomach, with 64% being found in the small bowel [8]. 

The histology of PJS-type hamartomatous polyps is characteristic for smooth muscle proliferation extending into the lamina propria with an arborization-like pattern formation. Hamartomatous polyps are usually considered benign [9]. But when it is associated with Peutz-Jegher syndrome, there is an elevated risk of malignancy in gastric and extra-intestinal sites [10]. Hearle et al. proposed an 85% risk of developing malignancy in patients with PJS by age 70 - a four-fold elevated risk compared to the general population [11]. However, the ability to extrapolate these findings to inform the risk of malignant transformation in solitary PJS remains unclear. While there are guidelines to inform the management and surveillance of PJS, there are currently no definitive guidelines for the management and surveillance of solitary PJS.

Definitive diagnosis of solitary PJS-type hamartomatous polyps depends on histopathological findings. Genetic analysis for mutations of the PJS susceptible gene, STK11/LB1 located at 19p13.3, as well as loss of heterozygosity at that locus have been used for the diagnosis of PJS [12].

## Conclusions

In conclusion, for patients diagnosed with solitary PJS-type hamartomatous polyps, it is recommended that a comprehensive evaluation of the signs and symptoms associated with PJS be completed for an accurate diagnosis to allow appropriate workup and management. This case poses a unique diagnostic dilemma in the management and surveillance of a solitary polyp of the small bowel with features of PJS and should serve to broaden the body of reported cases on the subject. 
